# Genome-wide analysis of the PreA/PreB (QseB/QseC) regulon of *Salmonella enterica *serovar Typhimurium

**DOI:** 10.1186/1471-2180-9-42

**Published:** 2009-02-23

**Authors:** Massimo Merighi, Alecia N Septer, Amanda Carroll-Portillo, Aditi Bhatiya, Steffen Porwollik, Michael McClelland, John S Gunn

**Affiliations:** 1 Center for Microbial Interface Biology and Department of Molecular Virology, Immunology and Medical Genetics, The Ohio State University, 333 W. 10thAvenue, Columbus, OH 43210, USA; 2 University of Texas Health Science Center at San Antonio, 7703 Floyd Curl Dr, San Antonio, TX 78229, USA; 3 Sidney Kimmel Cancer Center, 10835 Road to the Cure, La Jolla, CA 92121, USA; 4 1006 BRT, 460 W. 12th Avenue, Columbus, OH 43210, USA; 5 Department of Microbiology and Molecular Genetics, Harvard Medical School, Boston, MA 02115, USA; 6 Department of Biomolecular Materials and Interfaces, Sandia National Laboratories, Albuquerque, NM 87185, USA; 7 Department of Microbiology, University of Georgia, Athens, GA 30602, USA

## Abstract

**Background:**

The *Salmonella *PreA/PreB two-component system (TCS) is an ortholog of the QseBC TCS of *Escherichia coli*. In both *Salmonella *and *E. coli*, this system has been shown to affect motility and virulence in response to quorum-sensing and hormonal signals, and to affect the transcription of the *Salmonella enterica *serovar Typhimurium (*S*. Typhimurium) *pmrAB *operon, which encodes an important virulence-associated TCS.

**Results:**

To determine the PreA/PreB regulon in *S*. Typhimurium, we performed DNA microarrays comparing the wild type strain and various *preA *and/or *preB *mutants in the presence of ectopically expressed *preA *(*qseB*). These data confirmed our previous findings of the negative effect of PreB on PreA gene regulation and identified candidate PreA-regulated genes. A proportion of the activated loci were previously identified as PmrA-activated genes (*yibD*, *pmrAB*, *cptA*, etc.) or were genes located in the local region around *preA*, including the *preAB *operon. The transcriptional units were defined in this local region by RT-PCR, suggesting three PreA activated operons composed of *preA-preB*, *mdaB-ygiN*, and *ygiW*-STM3175. Several putative virulence-related phenotypes were examined for *preAB *mutants, resulting in the observation of a host cell invasion and slight virulence defect of a *preAB *mutant. Contrary to previous reports on this TCS, we were unable to show a PreA/PreB-dependent effect of the quorum-sensing signal AI-2 or of epinephrine on *S*. Typhimurium with regard to bacterial motility.

**Conclusion:**

This work further characterizes this unorthadox OmpR/EnvZ class TCS and provides novel candidate regulated genes for further study. This first in-depth study of the PreA/PreB regulatory system phenotypes and regulation suggests significant comparative differences to the reported function of the orthologous QseB/QseC in *E. coli*.

## Background

*Salmonella *spp. have a broad host range and antibiotic resistant isolates are on the rise [1]. Salmonellae infections of humans result in two primary clinical manifestations: enteric (typhoid) fever and gastroenteritis. The latter is characterized by a local infection primarily of the small intestine and involves massive neutrophil transmigration into the intestinal lumen. Typhoid fever is a systemic infection in which the bacterium is carried from the intestinal submucosa to distal organs primarily within host cells such as macrophages.

Two-component signal transduction is critical for the adaptation of *Salmonella enterica *serovar Typhimurium (*S*. Typhimurium) to the diverse array of environments encountered outside and inside its hosts [2]. These regulatory systems are typically composed of an inner membrane-bound sensor kinase (SK) and a cytoplasmic response regulator (RR). Environmental signals are often sensed by a periplasmic region of the SK, which then undergoes autophosphorylation followed by transfer of the phosphate to the RR. RR phosphorylation enhances DNA binding to recognition sites located in the promoters of regulated genes, subsequently activating or repressing transcription.

We recently described a novel *Salmonella *two-component system (TCS), PreA/PreB [3], which is similar to the quorum-sensing regulatory system QseB/QseC in enterohemorrhagic *Escherichia coli *[4]. PreB is a membrane-bound SK, with a periplasmic region containing a putative iron binding site (DxxE), while PreA is an OmpR-class RR. The *preAB *locus was identified in a transposon mutagenesis screen for regulators of *pmrCAB*, a locus encoding a separate TCS required for resistance to polymyxin B and itself part of the large PhoP/PhoQ TCS regulon. PreA activates by two-fold the transcription of *pmrCAB *in a PhoP- and PmrA- response regulator-independent fashion.

The signals controlling the PreA/PreB TCS are not known, and genetic evidence suggests that during growth in rich media, PreB primarily functions as a protein phosphatase inhibiting PreA function [3]. Curiously, the increase in *pmrCAB *transcription caused by PreA/PreB does not lead to observable transcriptional activation of most of the PmrA/PmrB regulon, with the exception of *yibD*, a putative glycosylase, nor does it lead to the alteration of the polymyxin resistance measured by MIC or time-to-death assays. Besides *pmrCAB *and *yibD*, no other targets of PreA/PreB are known [3], but the relatedness of *Salmonella *PreA/PreB to *E. coli *QseB/QseC suggested a potential wider role for this TCS.

The *E. coli *QseB/QseC TCS has been shown in various reports to sense quorum signal AI-3 as well as the eukaryotic hormones epinephrine/norepinephrine [5]. Activation of QseB/QseC results in the induction of flagellar gene synthesis and motility. Recently, while examining this TCS in *Salmonella *Typhimurium, bacterial motility was shown to increase in response to norepinephrine in the presence of iron [6]. Furthermore, *qseC *mutants were shown to possess virulence defects in rabbits (*E. coli *mutants) and pigs (*S*. Typhimurium mutants) [5,6].

In this work, we describe the use of DNA microarrays to explore the genome-wide transcriptional effects of non-polar mutations in *preA/preB *or of overexpression of the *preA *response regulator. These arrays corroborate previously published work relating to the role of PreB in regulated gene expression, identify several predicted PreA/PreB-regulated genes (many of which are located near *preAB*) and examine the role of this TCS in *Salmonella *pathogenesis.

## Methods

### Bacterial strains and media

*E. coli *and *S*. Typhimurium strains and plasmids used in this study are listed in Table 1[7-9]. Luria-Bertani (LB) broth and agar were used for strain maintenance, as well as cloning and expression experiments. When appropriate, antibiotics were added at the following concentrations: ampicillin, 100 μg/ml; kanamycin, 25 μg/ml; tetracycline, 15 μg/ml.

**Table 1:** Bacterial strains, plasmids and primers

Strains/Plasmids/Primers	Description	Source
*E. coli*		
DH5α	*supE44 *Δ(*lacZYA-argF*) *U169 *(Δ80*lacZ *Δ*M15*) *hsdR17 recA endA1 gyrA96 thi-1 relA1*	Gibco
*Salmonella enterica *serovar Typhimurium		
JSG210	ATCC 14208 (CDC6516-60), wild type	ATCC
JSG1998	JSG210 Δ*preA1998*	[3]
JSG2343	JSG210 Δ*preB2343*	[3]
JSG2626	JSG210 Δ*preAB2626*	[3]
JSG1225	*fliA*::Tn*10*dTet	gift of K. Klose
JSG648	*phoN*::cam *prgH1*::Tn*phoA*	[7]
**Plasmids**		
P_BAD_18	ColE1 *ori*, p*BAD *L-Ara inducible (Ap^r^)	[9]
pRK2013::Tn*7*	ColE1 *mob *Δ*tra*RK2 Δ*rep*RK2 *repE kan*::Tn*7 *(Tp^r ^Sm^r ^Sp^r^)	[8]
pJSG2558	P_BAD_18 with a 0.7-kb fragment containing *preA *expressed from pBAD (Ap^r^)	[3]
pJSG2581	P_BAD_18 with a 1.5-kb fragment containing *preAB *expressed from pBAD (Ap^r^)	[3]
**Primers (5'-3')**		
6-FAM-ccatcgccaataagtgtgtc	*preA *Reverse (primer ext.)	This study
6-FAM-cagggtgtcattcaactggc	*mdaB *Reverse (primer ext.)	This study
6-FAM-gatgacgctcaatgtggtcg	STM3175 Reverse (primer ext.)	This study
6-FAM-ttcgcaaactggtcgaggac	*ygiN *Reverse (primer ext.)	This study
6-FAM-tgatcacgtacatggagtag	*parC *Reverse (primer ext.)	This study
6-FAM-gtagaacacagtgccataac	*ygiW *Reverse (primer ext.)	This study
ggtagaacacagtgccataac	*preA *F (primer ext.)	This study
ctcggtaaaccagtcgacgc	*preA *R (primer ext.)	This study
ggaaggtggatttgaggc	*mdaB *F (primer ext.)	This study
gcagcttcaccgtcagagata	*mdaB *F (primer ext.)	This study
gacgatcttaacctgatgacc	*mdaB *R (primer ext.)	This study
cgaagtggataaagactggaac	STM3175 F (primer ext.)	This study
tagcgatagagcggaagc	STM3175 R (primer ext.)	This study
gcgtctatctgccattcc	*ygiN *F (primer ext.)	This study
gcggcatgatccaccatc	*ygiN *R (primer ext.)	This study
cctgaatttcgtccatgagg	*parC *F (primer ext.)	This study
gaatagcgagattcctggcg	*parC *F (primer ext)	This study
ccagctctgacatcgcatag	*parC *R (primer ext.)	This study
ccatcgccaataagtgtgtc	*ygiW *F (primer ext.)	This study
cgtcacgcagcgatttagc	*ygiW *R (primer ext.)	This study
ggccgaacactctttgtggt	*dnaN *F (real-time)	This study
gtataatttcggtcgcatccgt	*dnaN *R (real-time)	This study
atatcgtcgagcgcatttcc	*ygiW *F (real-time)	This study
tccagtctttatccacttcgcc	*ygiW *R (real-time)	This study
aagagttcgcgttgctggaa (JG1134)	*preA *F (real-time, RT-PCR)	This study
gagcttgcggcgtaaatgat	*preA *R (real-time)	This study
agactctggcgcctgactcg	*ygiN *F (real-time)	This study
aacgccggattccagaatacg	*ygiN *R (real-time)	This study
acaggcttaagagtagcggctg (JG1137)	*preB *R (RT-PCR)	This study
atatcgtcgagcgcatttcc (JG1132)	*ygiW *F (RT-PCR)	This study
cgcggatccttaacgaagcggcagatagatatc (JG1223)	STM 3175 R(RT-PCR)	This study
gtgtcgtttggcaacgccgcggaa (JG1703)	*preB *F(RT-PCR)	This study
caactggccgttggagtgcgcg (JG1704)	*mdaB *R (RT-PCR)	This study
tgccggatgttccgcgctataccgca (JG1705)	*mdaB *F (RT-PCR)	This study
tgacggtgatgttggcccggacgcg (JG1706)	*ygiN *R (RT-PCR)	This study
gaagccgtccagcagttg (JG1861)	STM 1595 F (Real-time PCR)	This study
gcgataaccattccaccaaac (JG1862)	STM 1595 R (Real-time PCR)	This study
cgttcctaaacttgcgttacag (JG1863)	STM 3175 F (Real-time PCR)	This study
gctggcgttgaccttatcc (JG1864)	STM 3175 R (Real-time PCR)	This study
ttgtatctggagattgtggactac (JG1865)	STM 1685 F (Real-time PCR)	This study
gagcccgtcgcaaagttg (JG1866)	STM 1685 R (Real-time PCR)	This study
tctacgcttgttcgcttac (JG1867)	STM 1252 F (Real-time PCR)	This study
ggtgttgtccagatattatgttc (JG1868)	STM 1252 R (Real-time PCR)	This study
tacagtggacaatgaatg (JG1869)	STM 1684 F (Real-time PCR)	This study
gctatggctatgtaacag (JG1870)	STM 1684 R (Real-time PCR)	This study
ggcttcacggcggcaatg (JG1871)	STM 2080 F (Real-time PCR)	This study
tcacgatacgggagggataaagg (JG1872)	STM 2080 R (Real-time PCR)	This study
ctaacttccaggaccactc (JG1873)	STM 4118 F (Real-time PCR)	This study
gataaccgtacagactcatac (JG1874)	STM 4118 R (Real-time PCR)	This study
tgatatgggcgttctggtctg (JG1875)	STM 1253 F (Real-time PCR)	This study
cgtgctgccagtgaggag (JG1876)	STM 1253 R (Real-time PCR)	This study

### Standard molecular biology and genetic techniques

DNA purification, molecular cloning, and PCR were performed following standard procedures [10]. Plasmids were mobilized by electroporation. Marked mutations were transferred between *S*. Typhimurium strains by P22 HT105 *int*-102 mediated generalized transduction as previously described [11]. Deletions were created previously using the lambda-Red procedure of Datsenko and Wanner [3,12]. DNA sequencing was performed using a Big Dye fluorescent terminator and an ABI3770 capillary sequencer at the Plant Microbe Genomic Facility (The Ohio State University).

### Microarray fabrication

Details of the construction of the backbone version of the *Salmonella *array were described previously [13]. PCR products were purified using the MultiScreen PCR 96-well Filtration System (Millipore, Bedford, MA), and eluted in 30 μl of sterile water. Subsequently, the products were dried, resuspended in 15 μl 50% DMSO, and 5 μl were rearrayed into 384-well plates for printing.

### Preparation of cDNA probes

A 0.5 ml overnight culture of *S. Typhimurium *was used to inoculate 10 ml of LB and grown at 37°C, with shaking to an OD_600 _of 0.6–0.7. When inducing ectopically expressed *preA *(or vector controls) with 10 mM arabinose, medium was buffered with 100 mM TrisHCl. Samples were transferred into chilled Falcon tubes containing 2 ml of 5% phenol/95% ethanol, incubated 15 min on ice, and cells were collected by centrifugation at 8000 g for 10 min at 4°C. Cells were lysed and RNA was collected, purified and DNase treated according to Promega SV Total RNA Isolation Kit (Promega, Madison, WI). RNA was checked for quantity and quality via gel electrophoresis or the Experion System (Bio-Rad, Hercules, CA). Cy3- and Cy5-dye-linked dUTP was directly incorporated during reverse transcription from total RNA to synthesize labeled cDNA probes, based on the method described by [13] with the following modifications: 15–100 μg of total RNA and 2.4 μg of random hexamers were resuspended in 30 μl of water, and subsequently the amounts and volumes of all components were doubled. Furthermore, 2 μl of RNase inhibitor (Invitrogen, Carlsbad, CA) was added to the reverse transcription, and the reaction incubated at 42°C for 2 h. After the first hour of incubation, 2 μl of additional Superscript II reverse transcriptase was added. Probes were purified using the QIAquick PCR purification kit (Qiagen, Valencia, CA) and eluted in 50 μl sterile water. Subsequently, probes were dried down to 20 μl final volume.

### Hybridization and data acquisition

Probes were mixed with equal volumes of 2× hybridization buffer containing 50% formamide, 10× SSC and 0.2% SDS, and boiled for 5 min. Probes were hybridized to the *Salmonella *array overnight at 42°C using a hybridization chamber (Corning, Corning, NY) submerged in water. Protocols suggested by the manufacturer for hybridizations in formamide buffer were applied for pre-hybridization, hybridization and post-hybridization wash processes. Scans were performed on an Affymetrix 428 Laser scanner (Affymetrix, Santa Clara, CA) using the Microarray suite 5.0 (Affymetrix) software.

### Data analysis

The TIFF files where unstacked using ImageJ (NIH) and signal intensities were quantified using the QuantArray 3.0 software package (Packard BioChip Technologies, Billerica, MA) at the Sidney Kimmel Cancer Center of San Diego. Unless noted otherwise, at least two slides (each containing triplicate arrays) were hybridized reciprocally to Cy3- and Cy5-labeled probes per experiment. Spots were analyzed by adaptive quantitation, and local background was subsequently subtracted from the recorded spot intensities. Ratios of the contribution of each spot to total signal in each channel were calculated (data normalization). Negative values (i.e., local background intensities higher than spot signal) were considered no data. The median of the six ratios per gene was recorded. For cDNA probes, ratios and standard deviations were calculated between the two conditions (e.g., experiment versus control). Genes with signals less than two standard deviations above background in both conditions were considered as not detected. The microarray data can be found at Gene Expression Omnibus http://www.ncbi.nlm.nih.gov/geo/ under series number GSE12866.

### Real time quantitative RT-PCR (qRT-PCR)

Two micrograms of RNA purified with the same protocol utilized for microarray analysis (but on different dates from different cultures) was used to synthesize cDNA using Invitrogen Superscript II in 25 μl reactions. Quantitative analysis of cDNAs and Ct value estimation was performed with an iCycler iQ5 system using SYBR Green I DNA binding dye (BioRad, Hercules, CA) to detect PCR products. The PCR mixture was prepared by mixing 12.5 μl 2X iQ SYBR Green, 0.5 μM of each primer (Table 1), and 50 ng of cDNA template. Parameters for the amplification were: initial denaturation at 95°C for 10 min, followed by 40 cycles each consisting of 15 s at 95°C, 30 s annealing at 55°C. The efficiency of amplification for each target gene was evaluated by calculating standard curves generated from 10-fold dilutions of each template sample followed by estimation using the regression model (Ct = m × Log(Dilution)+b). In all cases the efficiency ranged from 95 to 100%. Relative fold differences of gene expression between treatments were calculated using the 2^-ΔΔCt ^method with 16S rRNA or *dnaN *as standards. All qRT-PCR experiments were performed in triplicate at least twice with similar results.

### Operon transcript mapping by RT-PCR

Primers within the orfs for *preA*, *preB*, *mdaB*, *ygiN*, *ygiW*, and STM3175 were designed and used in RT-PCR reactions to determine if genes were co-transcribed. RNA from OD 0.6 cultures was isolated and cDNA was produced as described above. All RT-PCR experiments were performed on two separate occasions with cDNA derived from separate RNA preparations, each with similar results.

### Primer extension

Analysis of the 5' ends of mRNA transcripts was performed by primer extension as described by Merighi et al. 2006 [3]. 6-FAM-labeled primers (Table 1) and 50 μg cDNA were analyzed in an ABI 3770 capillary electrophoresis sequencer at the Plant Microbe Genomic Facility (The Ohio State University) along with DNA sequencing reactions using the same primer. In particular, Thermoscript was substituted for Superscript II (Invitrogen, Carlsbad, CA) and higher extension temperatures (65°C) were used to obviate secondary structure problems. All extension reactions were performed at least twice with independent RNA preparations and the reproducible peaks were selected.

### Animal cell cultures and invasion assay

HeLa cell lines were obtained from ATCC (Manassas, VA). Cells were grown to a monolayer at 37°C, 5% CO_2 _in DMEM with 10% heat-inactivated fetal bovine serum. Cells were then infected at an MOI of 100 in 24-well plates. Bacteria were spun onto the HeLa cells and incubated at 4°C for 30 min, then at 37°C for 1 hour. Extracellular bacteria were killed with 50 μg/ml gentamicin for 30 min. HeLa cells were then lysed with 0.1% Triton X-100 and plated for CFU determination.

### Mouse studies

Food and water were withdrawn 4 h before inoculation of female BALB/c mice (weighing 16 to 18 g). Mice (10 for each strain) were inoculated with 10^6 ^bacteria by oral gavage using a 22-gauge feeding needle. Dilutions of the stationary-phase cultures were plated to determine the number of bacteria present in the inoculum. For virulence assays, time of death was recorded as days post-infection. Competition infection experiments were conducted as described above, except that the mutant strain was co-infected with a chloramphenicol marked wild type strain (JSG224, *phoN2 ZXX*::6251dTn*10*-Cam). After plating bacteria on appropriate media from organs four days post-infection, the competitive index was calculated as the CFU mutant_plate count from organ_/wild type_plate count from organ _divided by mutant_inoculum_/wild type_inoculum_. All experiments were reviewed and approved by the Ohio State University Institutional Animal Care and Use Committee.

### Motility assays

0.3% agar DMEM plates were made containing, where indicated, 10 or 20 μM autoinducer-2 (AI-2 was a gift from Dr. Dehua Pei, Department of Chemistry, The Ohio State University), 10 or 50 μM epinephrine, or equivalent amounts of acidified water as a control for epinephrine plates (epinephrine was solubilized in acidified water). Overnight cultures were grown in LB, 37°C with shaking, adjusted to an OD of 0.1 at 600 nm and incubated for 2 hours at 37°C with shaking. Plates were stab-inoculated and incubated at 37°C for 14 hours. The diameter of the motility circles were measured at various times and compared.

## Results

### Transcriptome of the PreA/PreB two-component system

In previous experiments, we realized that the *preAB *TCS was not fully activated during growth in LB, as indicated by the absence of regulatory effects on the two known target genes (*yibD*, *pmrCAB*) when comparing a nonpolar mutation in the *preA *response regulator to the wild type strain [3]. This was confirmed in this study by microarray analysis co-hybridizing *preA *and wild type cDNA to a multistrain slide microarray of *Salmonella enterica *(data not shown). In our previous studies, transcriptional changes of the *preAB *target genes upon growth in LB was achieved by overexpression of PreA or by mutation of the sensor kinase *preB *[3]. We therefore analyzed the effect of overepressing PreA in a Δ*preA *strain carrying *preA *driven by a pBAD arabinose-inducible promoter grown in buffered LB. In addition, past experiments had implied that PreB may be acting as a protein phosphatase when bacteria are grown in LB [3]. If this is the case, some of the regulatory effects attributed to *preA *may have been dampened in the previous experimental design. We therefore proceeded to also analyze the cDNA from a *preAB *double mutant expressing pBAD-*preA *and a *preAB *strain carrying the vector control. All of the data from both experiments is included in Additional file 1, but a focused list of key candidate regulated genes is shown in Table 2.

**Table 2:** Microarray and real time PCR analysis showing a limited list of genes^a ^predicted to be PreAB activated

ORF	Gene	Function	Microarray A^b^ M^d ^(fold change)	Microarray B^c^ M (fold change)	qRT-PCR^e^
STM3707	*yibD*	putative glycosyltransferase	0.8 (1.7)	6.1 (68.6)	NP ^f^
STM3176	*ygiW*	Membrane protein (DUF388; exporter?)	4.5 (22.6)	5.2 (36.8)	355
STM1253		Cytochrome b561 (Ni^2+ ^dependent)	2.9 (7.5)	4.9 (29.9)	372
STM1595	*srfC*	*ssrAB *activated gene; predicted coiled-coil structure	4.3 (19.7)	4.7 (26.0)	1.2
STM3175		putative bacterial regulatory helix-turn-helix proteins, AraC family	3.6 (12.1)	4.4 (21.1)	605.3
STM1685	*ycjX*	putative ATPase	2.3 (4.9)	3.8 (13.9)	37.7
STM1252		putative cytoplasmic protein	1.5 (2.8)	2.8 (7.0)	8.6
STM3179	*mdaB*	NADPH specific quinone oxidoreductase (drug modulator)	1.0 (2.0)	2.8 (7.0)	32.5
STM1684	*ycjF*	putative inner membrane protein	1.1 (2.1)	2.6 (6.1)	61.2
STM4291	*pmrB*	sensory kinase in two-component regulatory system with PmrA	ND ^g^	2.1 (4.3)	NP
STM2080	*udg*	UDP-glucose/GDP-mannose dehydrogenase	ND	1.8 (3.5)	23.2
STM4292	*pmrA*	response regulator in two-component regulatory system with PmrB	ND	1.7 (3.2)	NP
STM4118	*yijP *(*cptA*)	putative integral membrane protein	ND	1.5 (2.8)	32.8
STM0628	*pagP*	PhoP-activated gene, palmitoyl transferase	ND	1.1 (2.1)	NP
STM2238		putative phage protein	0.9 (1.9)	1.0 (2.0)	NP

^a ^This list includes only those genes that were upregulated in both the *preA *and *preAB *mutant strains overexpressing *preA*, those confirmed by real-time PCR, genes previously shown to be preA-regulated (*yibD*, *pmrAB*) or those known to belong to the PhoPQ or PmrAB regulons

^b ^Δ*preA*/pBAD18-*preA *vs. Δ*preA*/pBAD18

^c ^Δ*preAB*/pBAD18-*preA *vs. Δ*preAB*/pBAD18

^d ^M = Log_2_(expression plasmid/vector control)

^e ^real time PCR (qRT-PCR) performed with cDNA derived from the strains used in Microarray B

^f ^NP = not performed

^g ^ND = not detected

Many of the genes upregulated in the Δ*preA *strain overexpressing *preA *(Table 2, column 1) were reconfirmed in experiments with the *preAB *mutant strain overexpressing *preA *(Table 2, column 2), but with increased fold activation. For instance, *yibD *was upregulated ~69-fold in the *preAB *mutant with pBAD-*preA *compared to the ~2-fold seen in PreB^+ ^backgrounds, while *mdaB *was upregulated ~7-fold versus 2-fold in the PreB^+ ^background. In addition, one of the discernable patterns from the two microarrays was that the three genes flanking the *preAB *operon: *ygiW*, STM3175, *mdaB*, were upregulated 37-, 21-, and ~7-fold, respectively (Table 2, column 2). Furthermore, in the *preAB *mutant background, we also observed upregulation of additional genes belonging to the PhoP/PhoQ and PmrA/PmrB regulons: *pmrAB*, *udg, cptA *(STM4118) and *pagP*. This further supports the connection between *preAB *and the two major regulons controlling genes involved in LPS modifications and antimicrobial peptide resistance in *Salmonella *and provides confidence to the quality of our microarray experiments.

### qRT-PCR analysis and transcriptional organization of *preAB *and flanking genes

To confirm the results of the microarray and to examine the regulation of *preAB *and the genes surrounding it, we performed qRT-PCR. The *preA *gene was shown to be induced 344-fold in a Δ*preB *strain vs. a wild type strain, furthering the previous finding of PreB acting primarily as a phosphatase when grown in LB and providing evidence of PreA-mediated positive autoregulation of *preAB*. The induction of *preB *in the microarray of the *preA *mutant background overexpressing *preA *also provided evidence of positive autoregulation of *preAB *(supplement Table 1). *ygiW *was strongly activated by PreA (355-fold) when comparing expression in a Δ*preAB*/pBAD18-*preA*^+^strain vs. Δ*preAB*/pBAD18. Using these same strains, *ygiN *was more weakly activated by PreA (2.94-fold). Several other PreA-regulated genes including STM3175 (605.3-fold) and *mdaB *(32.5-fold) were also analyzed by qRT-PCR, all confirming the regulation observed in the microarrays (though not always matching the observed fold-change) (Table 2).

**Figure 1:**
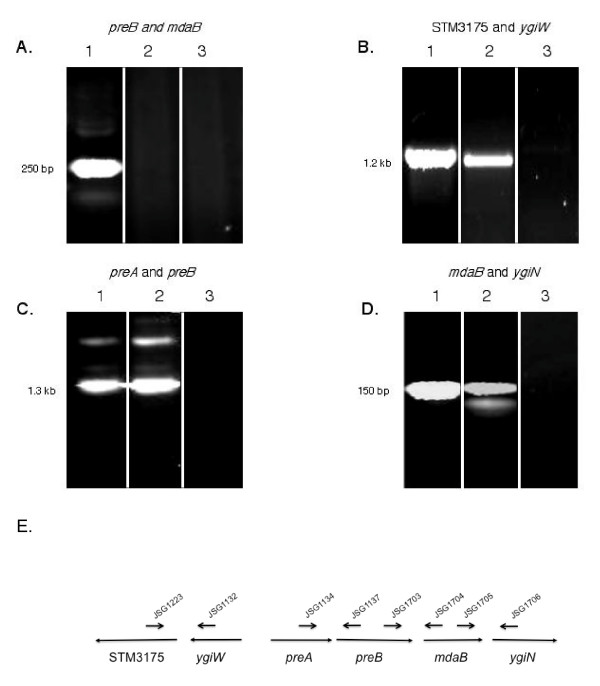
**Co-transcription analysis of the genes in the local chromosomal region surrounding *preA***. (A-D) The sets of genes examined are described above the ethidium bromide stained gels. The lane assignments in each set: (1) chromosomal DNA as a template; (2) cDNA as a template; (3) cDNA as a template, no reverse transcriptase. (E) A graphic representation of the *preA*-linked genes and the primers used for RT-PCR. The sequences for the primers can be found in Table 1.

The transcriptional organization of the *preAB *operon and of the genes flanking it, which were strongly upregulated by PreA, were analyzed by RT-PCR. As shown in Fig. 1, PCR fragments spanning *preA *and *preB*, *ygiW *and STM3175, and *mdaB *and *ygiN *were observed, suggesting that these sets of genes are co-transcribed. While primers spanning *preB *and *mdaB *(separated by a 106 bp intergenic region) yielded PCR product using a DNA template, no such product was observed with cDNA, even with the use of multiple primer sets, suggesting that these genes are not co-transcribed. These data, coupled with the microarray results, suggest that PreA is necessary for the activation of the *ygiW*-STM3175, *preA-preB*, and *mdaB-ygiN *operons.

**Figure 2:**
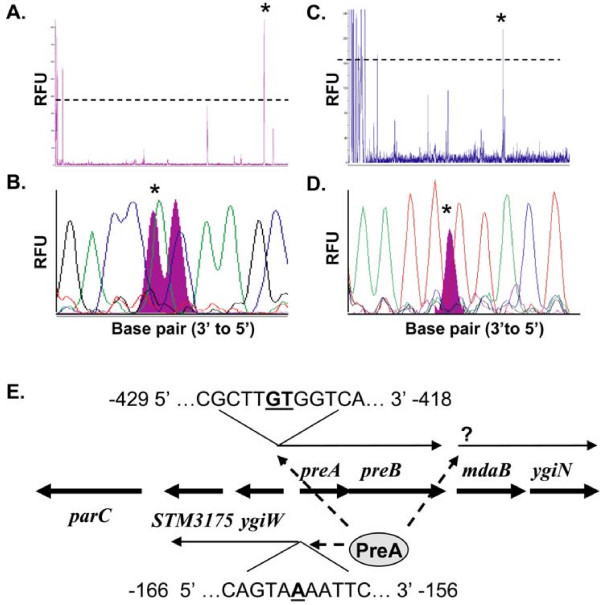
**Fluorescent primer extension analysis of transcriptional start sites for the *preAB *and *ygiW*-STM3175 operons**. Electropherograms of the labeled cDNA are shown for *preA *(A) and *ygiW *(C). Dashed lines mark the relative fluorescence unit (RFU) cut-off, below which does not give a confident signal strength. Asterisks (*) denote which cDNA peak was analyzed. Labeled cDNA electropherograms (filled peaks) were aligned with sequence chromatograms (open peaks) to identify the base at which transcription starts for both *preAB *(B) and *ygiW*-STM3175 (D). Results of transcriptional organization are diagramed as shown with start sites mapped relative to the translational start (E). PreA appears to activate transcription of each of the three operons defined in the *preA *region (dashed lines denote positive regulation).

We next attempted to map the transcriptional start sites of these three operons by primer extension using a fluorescent primer protocol. Using this approach, the start of transcription for the *preAB *operon was identified at -423/424 bp from the start codon, implying that the *preAB *promoter is internal to *ygiW *and contains a large, untranslated leader region (Fig. 2). The start site of the *ygiW*-STM3175 operon was at -161 bp, which is 10 bp internal to the *preA *open reading frame. Multiple attempts were made to map the *mdaB-ygiN *start, however we were unsuccessful at identifying a clear site for transcriptional initiation.

### Phenotypes of *preAB *TCS mutants

**Figure 3:**
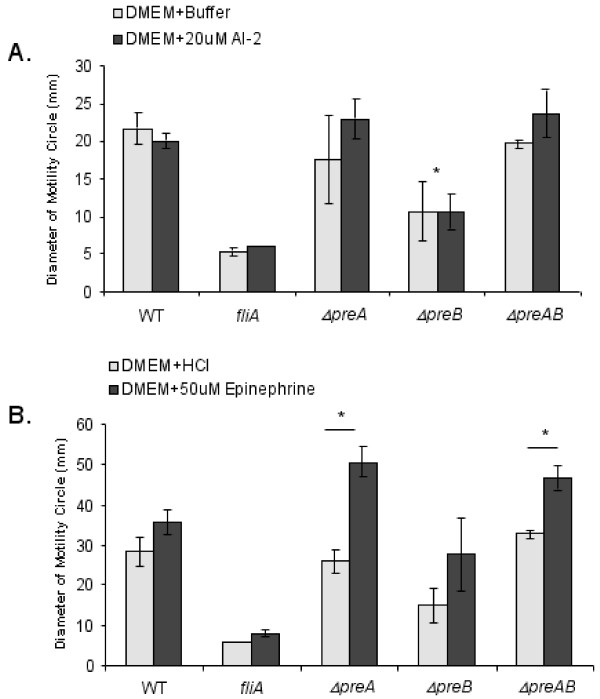
**Motility assays were performed using DMEM soft agar plates**. Motility ring diameters of the wild type 14028s strain and a negative control (*fliA*) were compared to *preA*, *preB*, and *preAB *strains. Signaling molecules were tested for possible affects on motility. (A) 20 μM AI-2 (dark bars) or an equal volume of buffer (light bars) were added to the medium. (B) 50 μM epinephrine (dissolved in acidified water, dark bars) or an equal volume of acidified water (light bars) was added to medium. An asterisk (*) denotes statistical significance with a p-value < 0.02 as determined with a student t-test. The asterisk in (A) is in comparision of Δ*preB *to the wild type strain.

We previously reported that PreA/PreB is orthologous to the *E. coli *QseBC system, which responds to AI-3 and epinephrine/norepinephrine signals. In response to these signals, the QseC sensor kinase has been reported to affect motility in both *E. coli *and *S*. Typhimurium [6,14]. However, our microarray data did not suggest any major and/or consistent effect of PreA/PreB on transcription of the flagellar operon. Therefore, we assessed the effects of mutations in *preA *and *preB *on the motility of *S*. Typhimurium on agar plates with DMEM as the culture medium. The results showed a reduction in motility for the *preB *sensor mutant (Fig. 3) but not for the *preA *or *preAB *mutants. As seen with QseC in *E. coli*, the addition of synthetic AI-2 did not complement the *preB *mutant motility defect and also did not affect the motility of the wild type strain (Fig. 3A). Additionally, though epinephrine/norepinephrine has been reported to activate motility in both *E. coli *and *S*. Typhimurium [6,15], a slight but non-significant increase in wild type strain motility was observed in our assays using identical conditions and epinephrine concentrations used previously in *E. coli*. Supplementation of the media with epinephrine did increase the motility of *preA*, *preB *and *preAB *mutants (all statistically significant except *preB*, Fig. 3B), but as this effect of epinephrine on *S*. Typhimurium motility was observed only in *preA or preB *mutant strains, this effect is not mediated by PreA/PreB.

Overexpression of *mdaB *[16] and mutation of *preB *(*ygiY*; [17]) were previously shown to affect drug resistance in *E. coli *and oxidative stress response in *Helicobacter *spp. [18-20]. In addition, catalase genes appear PreA-regulated (Additional file 1). *preAB *mutant strains were therefore analyzed for resistance to various chemicals and antibiotics, including nalidixic acid, pyrazinoic acid, H_2_O_2_, paraquat, adriamycin, and tetracycline. None of the mutants showed increased sensitivity when compared to the wild type strain (data not shown).

**Figure 4:**
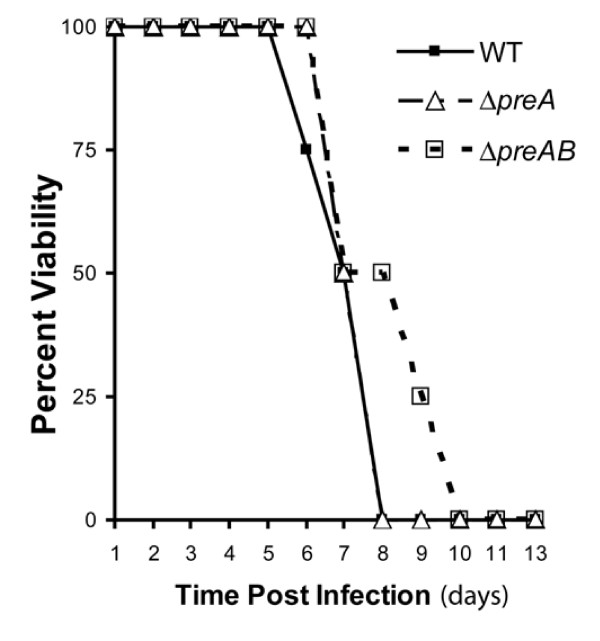
**Female BALB/c mice were inoculated with 10^6 ^bacteria via oral gavage and animals were monitored over a period of 13 days**.

To determine if the PreA/PreB system affects virulence, mutant and wild type strains were perorally inoculated in mice and mortality was recorded over two weeks. The *preA *mutant showed no virulence defect while mice infected with the *preAB *strain showed a consistent two day delay in mortality, but eventually all mice succumbed to infection (Fig. 4). The *preAB *mutant strain also demonstrated a consistent competition infection defect (competitive index: spleen, 0.344; liver, 0.326) when co-inoculated by oral gavage with the wild type strain, which was not observed with strains containing single mutations in *preA *or *preB *(data not shown). Thus, the PreA/PreB TCS has a slight but reproducible effect on virulence in mice.

**Figure 5:**
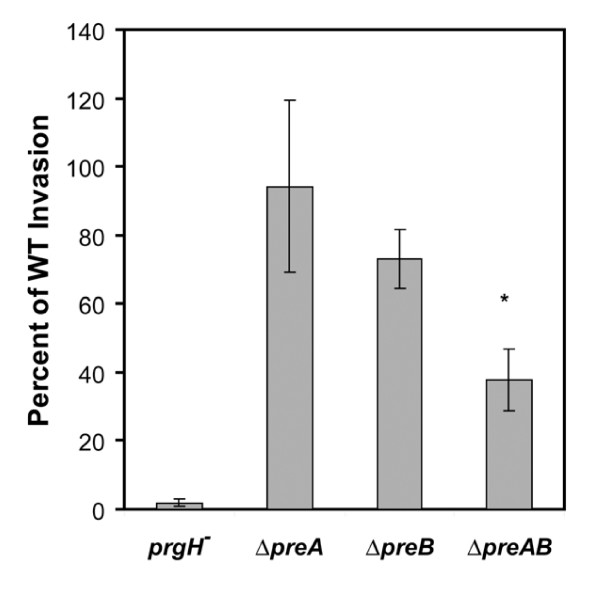
**HeLa cell invasion assays were performed for wild type, *prgH *(negative control), *preA*, *preB*, and *preAB *strains**. HeLa cells were grown to monolayer in DMEM with 10% FBS at 37°C and 5% CO_2_. Cells were then infected with bacteria at an MOI of 100 in 24-well plates. Data is presented as percent of wild type CFUs. The asterisk (*) denotes statistical significance with a p-value of < 0.02 as determined with a student t-test.

Given that invasion of the small intestine is a prerequisite to systemic infection upon oral inoculation, we also evaluated the ability of various *preA/preB *mutants to invade HeLa cells grown in vitro. Again, the response regulator (*preA*) mutant did not show any defect in invasion of HeLa cells. The *preB *strain showed a marginal and non-significant reduction in invasion upon 2 hours co-cultivation at a MOI = 100 (invasion ~80% of wild type), while a larger defect was observed for the *preAB *double mutant (~30% of WT) (Fig. 5). Therefore, the PreA/PreB TCS has a direct or indirect effect on host cell invasion.

## Discussion

TCSs are important for bacterial survival in host and non-host conditions. We previously identified a TCS (PreA/PreB/QseB/QseC) that indirectly affected the transcriptional activation of the PmrA/PmrB TCS of *Salmonella *[3]. Some of the genes of the PmrA/PmrB regulon were affected by PreA/PreB, but antimicrobial peptide resistance mediated by PmrA/PmrB was unaffected by the presence of PreA/PreB. Because we had few clues to the potential function of this TCS in *Salmonella*, we pursued a microarray approach to identify regulated genes that might suggest phenotypes related to PreA/PreB.

Previous research demonstrated that PreB acts preferentially in laboratory growth media (e.g. LB) in a negative manner with regard to PreA gene regulation- likely acting as a phosphatase leaving PreA unphosphorylated and inactive. We have not yet identified a growth condition where this is not the case. These observations also held true with the microarray analysis, as we observed more regulated genes and a higher level of regulation in the absence of PreB than in its presence. This was true even when PreA was overexpressed. Thus, in the absence of known environmental conditions that activate this TCS, the strain expressing the most PreA-regulated loci is one in which PreA is overexpressed in the absence of PreB.

Comparison of the results of two microarray analyses, (*preA *mutant/p*preA *[PreA overexpressed] vs. *preA *mutant with empty vector; *preAB *mutant/p*preA *[PreA overexpressed] vs. *preAB *mutant with empty vector), showed reasonable agreement, with about 40% of the genes in the *preA *mutant background array also observed in the *preAB *mutant background array (Additional file 1; Table 2). There were few candidate repressed loci but these were more numerous than the activated genes in the *preAB *mutant p*preA *vs. *preAB *mutant with empty vector arrays. If our model concerning the phosphatase function of PreB is accurate, this may suggest that phosphorylation of PreA is required for it to act as a repressor.

The repressed and activated genes in the Additional file 1 and Table 2 show little commonality, except the presence of known PmrA-regulated genes [STM3707 (*yibD*), STM1252/53, STM4292 (*pmrA*), STM4291 (*pmrB*), STM2080 (*ugd/pmrE*), and STM4118 (*yijP/cptA*)] and genes in the local region around *preA *[STM3177 (*preA*), STM 3178 (*preB*; from Table 2), STM3176 (*ygiW*), STM 3175, and STM 3179 (*mdaB*)]. We further analyzed the transcriptional units located in the vicinity of *preA*, showing that the PreA- activated operons were composed of *preA-preB*, *mdaB-ygiN*, and *ygiW*-STM3175. *preB *and *mdaB *were not shown by RT-PCR to be co-transcribed. The operonic arrangement of *preA *and *preB *and the activation of this operon by PreA are in agreement with the study of *qseBC *in enterohemorrhagic *E. coli *(EHEC) ([21]).

Transcriptional initiation sites were defined for *preA-preB *and *ygiW*-STM3175 but not *mdaB-ygiN*. The *preAB *start site does not match those mapped for *qseBC *in EHEC, which occur at -27 and -78 with respect to the *qseB *ATG. However, QseB binds to the EHEC *qseBC *promoter near its transcriptional starts (-27 to -40) but also in a region (-409 to -423) that is located near the transcriptional initiation site we mapped for *preAB *[21]. We hypothesize that PreA binds to the promoter region of each of these operons (*preA-preB*, *mdaB-ygiN*, and *ygiW*-STM3175) to activate transcription, and future work will define the PreA binding sites in these regulated promoters.

It has been previously demonstrated that QseC (PreB ortholog) of EHEC is a receptor for host-derived epinephrine/norepinephrine and intestinal flora derived AI-3 [5]. In *E. coli*, QseB positively regulates the transcription of flagellar genes and thus flagellar synthesis and motility. *S*. Typhimurium motility has also been shown to be affected by norepinephrine and QseC/PreB [6]. However, we were unable to demonstrate a role of PreA/PreB in the regulation of flagellar genes or a role for PreA/PreB in motility, except for an effect of a *preB *mutation alone. Furthermore, the addition of AI-2 or epinephrine had no effect on wild type motility. Epinephrine did surprisingly increase motility of *preA *and *preAB *mutants, but this effect was clearly PreA/PreB independent. Recently, Bearson et al. [22] demonstrated that norepinephrine acts as a siderophore, and that mutations affecting iron transport no longer responded to norepinephrine. Thus it remains a strong possibility that any effects observed on bacteria by epinephrine/norepinephrine are due to enhanced iron availability. PreB contains a putative iron binding motif in its periplasmic region, thus furthering a presumed association of iron with the regulation of PreA/PreB.

Though PreA/PreB regulates genes that affect antimicrobial peptide resistance (*pmrAB*, *cptA*) and resistance to a variety of drugs (*mdaB*) or reactive oxygen compounds (e.g. *katE*, STM1731, *dps*), none of the *preA *or *preB *mutations affected antimicrobial susceptibility. However, the loss of both *preA *and *preB *affected both invasion of epithelial cells in vitro (though no consistant effect of PreA/B on *Salmonella *Pathogenicity island 1 invasion genes was observed) and virulence in the mouse model. Future work will focus on genes regulated by PreA/PreB that contribute to these phenotypes.

## Conclusion

PreA/PreB is a TCS that regulates *Salmonella *genes including those of the PmrA/PmrB regulon and those adjacent to *preAB *on the chromosome. RNA analysis of the genes surrounding *preA *revealed three PreA-activated operons composed of *preA-preB*, *mdaB-ygiN*, and *ygiW*-STM3175. Though PreA/PreB do not appear to be responsive to host-derived hormones or microbial quorum-sensing signals as has been previously reported, PreA/PreB do play a role in *Salmonella *host cell invasion and virulence.

## Authors' contributions

AB performed RT-PCR and other RNA experiments. AC-P perfomed the initial work with this TCS and constructed some of the mutant strains. SP and MMc constructed the arrays and performed the microarray statistical analysis. MMc aided in the final preparation of the manuscript. ANS and MM together perfomed microarray analysis and all other experiments, and jointly wrote the first draft of the manuscript. JSG participated in the writing of the manuscript, the interpretation of the data, and conceived the study. All authors read and approved the final version of the manuscript.

## Supplementary Material

Additional file 1 **Candidate PreA-regulated genes identified by microarray analysis.** This table is a complete list of candidate PreA-regulated genes identified by microarray analysis of RNA isolated from strains overexpressing *preA *(in *preA *[Microarray A] and *preAB *[Microarray B] mutant backgrounds). Click here for file
