# Supplementary material for: Genome-wide analysis of the PreA/PreB (QseB/QseC) regulon of Salmonella enterica serovar Typhimurium
Source: BMC Microbiol. 2009 Feb 23;9:42. doi: 10.1186/1471-2180-9-42 (PMC2653508; doi:10.1186/1471-2180-9-42)
Supplement: Additional file 1 — Candidate PreA-regulated genes identified by microarray analysis. This table is a complete list of candidate PreA-regulated genes identified by microarray analysis of RNA isolated from strains overexpressing preA (in preA [Microarray A] and preAB [Microarray B] mutant backgrounds). [file 1471-2180-9-42-S1.doc]

**Table S1.** Candidate PreA-regulated genes identified by microarray analysis of RNA isolated from strains overexpressing *preA* (in *preA* [Microarray A] and *preAB* [Microarray B] mutant backgrounds)

ORF Gene Function Microarray A^a^ Microarray B^b^

M^c^ (fold change) M (fold change)

STM3707 *yibD* putative glycosyltransferase 0.8 (1.8) 6.1 (68.9)

STM3176 *ygiW* Membrane protein (DUF388; exporter?) 4.5 (23.0) 5.2 (35.7)

STM1253 Cytochrome b561 (Ni^2+^ dependent) 2.9 (7.6) 4.9 (30.0)

STM1595 *srfC* *ssrAB* activated gene: predicted coiled-coil structure 4.4 (20.4) 4.7 (26.2)

STM3175 putative bacterial regulatory helix-turn-helix proteins, AraC family 3.6 (12.3) 4.4 (21.7)

STM1685 *ycjX* putative ATPase 2.3 (4.9) 3.8 (14.2)

STM1252 putative cytoplasmic protein 1.5 (2.8) 2.8 (7.1)

STM3179 *mdaB* NADPH specific quinone oxidoreductase (drug modulator) 1.0 (2.0) 2.8 (7.0)

STM3178 *preB* sensory histidine kinase in regulatory system 2.8 (7.1) ND^d^

STM0865 *ybjG* putative permease ND 2.7 (6.5)

STM1684 *ycjF* putative inner membrane protein 1.1 (2.1) 2.6 (6.0)

STM4293 *yjdB* putative integral membrane protein ND 2.4 (5.1)

STM0802 *moaA* molybdopterin biosynthesis, protein A ND 2.2 (4.6)

STM4291 *pmrB* sensory kinase in two-component regulatory system with BasR ND 2.1 (4.2)

STM2330 *lrhA* NADH dehydrogenase transcriptional repressor (LysR family) ND 2.1 (4.2)

STM2080 *udg* UDP-glucose/GDP-mannose dehydrogenase ND 1.8 (3.4)

STM3678 putative bacterial regulatory helix-turn-helix proteins, AraC family ND 1.7 (3.4)

STM4292 *pmrA* response regulator in two-component regulatory system with BasS ND 1.7 (3.3)

STM0803 *moaB* molybdopterin biosynthesis, protein B ND 1.7 (3.2)

STM3414 *rplQ* 50S ribosomal subunit protein L17 1.7 (3.2) ND

STM4118 *yijP* putative Integral membrane protein ND 1.5 (2.9)

STM1960 *fliD* flagellar biosynthesis; filament capping protein; enables filament assembly 1.5 (2.9) ND

STM3706 *yigQ* putative periplasmic protein ND 1.4 (2.7)

STM0804 *moaC* molybdopterin biosynthesis, protein C ND 1.4 (2.6)

STM3174 *parC* DNA topoisomerase IV, subunit A ND 1.1 (2.2)

STM0628 *pagP* PhoPQ-activated gene ND 1.1 (2.1)

STM0611 putative oxidoreductase protein ND 1.0 (2.0)

STM4597 putative periplasmic protein ND 1.0 (2.0)

STM2238 putative phage protein 0.9 (1.8) 1.0 (2.0)

STM3576 *zntA* P-type ATPase family, Pb/Cd/Zn/Hg transporting ATPase ND 0.9 (1.9)

STM3006 *ygdQ* putative integral membrane transport protein ND 0.9 (1.9)

STM0953 *infA* protein chain initiation factor IF-1 1.0 (2.0) ND

STM4150 *rplA* 50S ribosomal subunit protein L1, regulates synthesis of L1 and L11 1.0 (2.0) ND

STM0216 *rpsB* 30S ribosomal subunit protein S2 0.9 (1.9) ND

STM3911 putative inner membrane protein 0.9 (1.9) ND

STM2587 Gifsy-1 prophage: similar to phage tail assembly protein 0.9 (1.9) ND

STM3384 *yhdG* putative TIM-barrel enzyme, possibly dehydrogenase 0.9 (1.9) ND

STM4466 putative carbamate kinase 0.9 (1.9) ND

STM3209 *rpsU* 30S ribosomal subunit protein S21 0.9 (1.9) ND

STM3288 *yhbC* putative cytoplasmic protein 0.9 (1.8) ND

STM2238 putative phage protein 0.9 (1.8) ND

STM1029 Gifsy-2 prophage 0.9 (1.8) ND

STM0837 *ybiS* putative periplasmic protein 0.8 (1.8) ND

STM1187 *rluC* 23S rRNA pseudouridylate synthase 0.8 (1.8) ND

STM0248 *yaeD* putative dehydratase 0.8 (1.8) ND

STM1054 Gifsy-2 prophage 0.8 (1.8) ND

STM1731 putative catalase ND -1.9 (3.9)

STM1729 *yciF* putative cytoplasmic protein ND -1.8 (3.4)

STM1559 putative glycosyl hydrolase ND -1.7 (3.2)

STM1563 *osmC* putative resistance protein, osmotically inducible -0.9 (1.9) -1.7 (3.2)

STM0361 cytochrome BD2 subunit II ND -1.5 (2.9)

STM3157 *yghA* putative oxidoreductase ND -1.5 (2.8)

STM1928 *otsA* trehalose-6-phosphate synthase ND -1.5 (2.8)

STM0360 cytochrome BD2 subunit I ND -1.5 (2.8)

STM1311 *osmE* transcriptional activator of ntrL gene ND -1.5 (2.8)

STM4240 *yjbJ* putative cytoplasmic protein ND -1.4 (2.7)

STM4242 putative outer membrane or exported ND -1.4 (2.7)

STM0935 *poxB* pyruvate dehydrogenase/oxidase ND -1.4 (2.6)

STM1318 *katE* catalase; hydroperoxidase HPII(III), RpoS-dependent ND -1.4 (2.6)

STM1929 *otsB* trehalose-6-phosphate phophatase, biosynthetic ND -1.3 (2.5)

STM1285 *yeaG* putative Ser protein kinase ND -1.3 (2.5)

STM3909 *ilvC* ketol-acid reductoisomerase -1.3 (2.5) ND

STM2141 *fbaB* 3-oxoacyl-[acyl-carrier-protein] synthase I -1.0 (2.1) -1.3 (2.4)

STM4561 *osmY* hyperosmotically inducible periplasmic protein, RpoS-dependent ND -1.3 (2.4)

STM1558 putative glycosyl hydrolase ND -1.3 (2.4)

STM4519 putative NAD-dependent aldehyde dehydrogenase ND -1.2 (2.4)

STM1796 *treA* trehalase, periplasmic ND -1.2 (2.4)

STM0831 *dps* stress response DNA-binding protein; starvation induced resistance to H_2_O_2_ ND -1.2 (2.3)

STM0359 putative cytoplasmic protein ND -1.2 (2.3)

STM3648 *yiaG* putative transcriptional regulator ND -1.2 (2.3)

STM2474 *tktB* transketolase 2, isozyme ND -1.1 (2.2)

STM2165 *yehZ* putative ABC superfamily transport protein ND -1.1 (2.2)

STM1932 *ftnB* ferritin-like protein ND -1.1 (2.2)

STM1804 *ycgB* putative cytoplasmic protein ND -1.1 (2.1)

STM3228 *yqjC* putative periplasmic protein ND -1.1 (2.1)

STM_PSLT063 putative cytoplasmic protein ND -1.1 (2.1)

STM0308 *yafV* putative amidohydrolase -1.0 (2.0) ND

STM4240 *yjbJ* putative cytoplasmic protein ND -1.0 (2.0)

STM4486 *yjgB* putative alcohol dehydrogenase ND -1.0 (2.0)

STM3538 *glgB* 1,4-alpha-glucan branching enzyme ND -1.0 (2.0)

STM1491 ABC-type proline/glycine betaine transport systems, ATPase component ND -1.0 (2.0)

STM3537 *glgX* glycosyl hydrolase ND -1.0 (2.0)

STM1588 *yncC* putative regulatory protein, GntR family ND -1.0 (2.0)

STM4290 *proP* MFS family, low-affinity proline transporter (proline permease II) ND -1.0 (2.0)

STM3231 *yqjK* putative inner membrane protein ND -0.9 (1.9)

STM1560 putative alpha amylase ND -0.9 (1.9)

STM1589 *yncB* putative NADP-dependent oxidoreductase ND -0.9 (1.9)

STM3967 *dlhH* putative dienelactone hydrolase family ND -0.9 (1.9)

STM3067 *yggB* putative membrane protein ND -0.9 (1.9)

STM2473 *talA* transaldolase A ND -0.9 (1.9)

STM0169 *gcd* glucose dehydrogenase ND -0.9 (1.8)

^a^  ∆*preA*/pBAD18-*preA* vs. ∆*preA*/pBAD18

^b^  ∆*preAB*/pBAD18-*preA* vs. ∆*preAB*/pBAD18

^c^ M=Log_2_(expression plasmid/vector control)

^d^ ND= not detected above 1.8 fold cutoff
